# Neuropeptides encoded by *nlp-49* modulate locomotion, arousal and egg-laying behaviours in *Caenorhabditis elegans* via the receptor SEB-3

**DOI:** 10.1098/rstb.2017.0368

**Published:** 2018-09-10

**Authors:** Yee Lian Chew, Laura J. Grundy, André E. X. Brown, Isabel Beets, William R. Schafer

**Affiliations:** 1 Neurobiology Division, MRC Laboratory of Molecular Biology, Cambridge CB2 0QH, UK; 2 Cell Biology Division, MRC Laboratory of Molecular Biology, Cambridge CB2 0QH, UK; 3 MRC London Institute of Medical Sciences, London W12 0NN, UK; 4 Institute of Clinical Sciences, Imperial College London, London W12 0NN, UK; 5 Department of Biology, KU Leuven, 3000 Leuven, Belgium

**Keywords:** *Caenorhabditis elegans*, neuropeptides, locomotion, arousal, egg-laying

## Abstract

Neuropeptide signalling has been implicated in a wide variety of biological processes in diverse organisms, from invertebrates to humans. The *Caenorhabditis elegans* genome has at least 154 neuropeptide precursor genes, encoding over 300 bioactive peptides. These neuromodulators are thought to largely signal beyond ‘wired’ chemical/electrical synapse connections, therefore creating a ‘wireless’ network for neuronal communication. Here, we investigated how behavioural states are affected by neuropeptide signalling through the G protein-coupled receptor SEB-3, which belongs to a bilaterian family of orphan secretin receptors. Using reverse pharmacology, we identified the neuropeptide NLP-49 as a ligand of this evolutionarily conserved neuropeptide receptor. Our findings demonstrate novel roles for NLP-49 and SEB-3 in locomotion, arousal and egg-laying. Specifically, high-content analysis of locomotor behaviour indicates that *seb-3* and *nlp-49* deletion mutants cause remarkably similar abnormalities in movement dynamics, which are reversed by overexpression of wild-type transgenes. Overexpression of NLP-49 in AVK interneurons leads to heightened locomotor arousal, an effect that is dependent on *seb-3.* Finally, *seb-3* and *nlp-49* mutants also show constitutive egg-laying in liquid medium and alter the temporal pattern of egg-laying in similar ways. Together, these results provide *in vivo* evidence that NLP-49 peptides act through SEB-3 to modulate behaviour, and highlight the importance of neuropeptide signalling in the control of behavioural states.

This article is part of a discussion meeting issue ‘Connectome to behaviour: modelling *C. elegans* at cellular resolution’.

## 1. Background

Neuropeptides regulate a myriad of behaviours in organisms ranging from invertebrates to humans [1–5]. Understanding how neuropeptide signalling between neurons drives different motor outputs not only provides important information on how behavioural states come about, but also provides insight into how neuromodulators interact with the physical (wired) connectome to drive such changes. There is increasing evidence that neuropeptides act on neuronal circuits to promote the functional importance of particular connections over others [6,7]. *Caenorhabditis elegans* provides an excellent system to study the role of neuropeptides in regulating behaviour owing to its ease of genetic manipulation and availability of a complete wiring diagram for its compact nervous system [8], providing a unique opportunity to investigate neuropeptide signalling in the context of the wired connectome in a whole organism.

The *C. elegans* genome encodes 154 predicted neuropeptide precursor genes, which can be processed to over 300 different peptides [9,10], of which at least 200 have been confirmed by mass spectrometry [11]. Neuropeptides are important signalling molecules formed from larger, inactive precursors that are processed enzymatically to produce bioactive peptides [1,12,13]. They have been implicated in a wide variety of biological processes including locomotion, sensory responsiveness, egg-laying and quiescence (reviewed in [1,14]). In *C. elegans* and other organisms, neuropeptides primarily exert their effects via G protein-coupled receptors (GPCRs) [5]. Based on phylogenetic analysis, GPCRs can be classified into Glutamate, Rhodopsin, Adhesion, Frizzled/Taste2 and Secretin families [15]. The Secretin family (or family B) is one of the main receptor families for neuropeptides and neurohormones, several of which have been implicated in important biological functions [16]; for example, corticotrophin-releasing factor (CRF), which modulates anxiety and stress in humans [17].

Here, we investigated how behavioural states are affected by signalling through the *C. elegans* neuropeptide receptor SEB-3, a member of an orphan secretin receptor family that had already evolved in a common ancestor of bilaterian animals [5]. Using reverse pharmacology, we identified NLP-49 as a neuropeptide ligand of this ancient GPCR family. We found that NLP-49 peptides released from only a few head neurons can affect multiple motor outputs through a receptor that is broadly expressed throughout the nervous system. Specifically, our data represent the first description, to our knowledge, of a neuropeptide ligand for the SEB-3 family of secretin receptors in bilaterian animals and the biological functions of this newly identified ligand–receptor system, demonstrating a role for NLP-49 peptides and SEB-3 in arousal and egg-laying behaviours.

## 2. Experimental procedures

### (a) Strain and transgene information

Strains were maintained on nematode growth medium (NGM) plates seeded with *E. coli* strain OP50 according to standard experimental procedures [18]. Day 1 adult hermaphrodite animals were used for all experiments. Where indicated, mutant alleles were backcrossed 4–6 times to our laboratory stock of N2 (wild-type). For a list of strains and transgene details, see electronic supplementary material, table S1. For most experiments, three independent *nlp-49* overexpression lines were tested, and data for one (AQ3837: *ljEx1004*[P*nlp-49*::*nlp-49*::*SL2-mKate2*;*unc-122*::*gfp*]) is shown. One of these lines was integrated (AQ4044: *ljIs152*[P*nlp-49*::*nlp-49*::*SL2-mKate2*;*unc-122*::*gfp*]) and used for tap arousal experiments. Transgenes were cloned using the multisite gateway three-fragment cloning system (12537-023, Thermo Fisher Scientific) into pDESTR4R3 II. For transgenic reporter lines described here, the length of the promoter (number of bases before ATG) for each gene is as follows: *H05L03.3/nlp-49* 2019 bases; *C18B12.2/seb-3* 2014 bases. For the list of reporter transgenic lines used to confirm expression patterns, see electronic supplementary material, table S2. Other promoters used for heterologous expression are P*flp-1=* 1571 bp, P*flp-12=* 2652 bp, P*egl-6=* 3527 bp and P*cat-1* = 2346 bp.

### (b) Behavioural tests

#### (i) Egg-laying assays

Video recordings of animals freely moving on food was performed using WormTracker 2.0 software [19,20] and analysed using a custom matlab (MathWorks) script as described in [21,22]. At least 54 h of video obtained for multiple days was analysed per genotype (table 1), where an individual worm was recorded for 6 h. Animals were tracked on multiple days. Egg-laying behaviour off-food was performed oby moving well-fed day 1 adult worms onto low-peptone NGM plates seeded (overnight) with 20 µl OP50 or unseeded, and eggs laid counted after 60 min. For this experiment, 12 animals were tested per condition (on food or off food) for each genotype, in at least three replicates. Egg-laying in liquid was carried out in CTX buffer (5 mM KH_2_PO_4_/K_2_HPO_4_ (pH 6), 1 mM CaCl_2_ and 1 mM MgSO_4_) with six technical replicates per genotype (per biological replicate) and 10 animals per technical replicate, with eggs counted after 60 min. At least three biological replicates were conducted. Animals were transferred to 1 ml CTX buffer in each well of a 24-well plate using a mounted hair pick. Egg retention experiments were performed on animals aged 16 h post larval stage four (L4) and eggs counted using bright-field microscopy. At least three biological replicates were conducted with 15–20 animals tested per replicate. Egg retention experiments were conducted blind to the genotype of the strains tested.

**Table 1.** Egg-laying parameters. Probability (*p*) refers to the probability of the animal laying an egg during the active state. The intra-cluster time (s) refers to the time between egg-laying events during the active state. The inter-cluster time (min) refers to the time between active states, i.e. duration of the inactive state. The duration of the active phase (s) is inferred from *p* and the intra-cluster time constant. Values are provided as mean ± s.e.m. Video (h) indicates the number of hours of video analysed to extract parameters for analysis. Statistical differences were determined using an unpaired *t*-test. n.s., not significant, ** < 0.01, *** < 0.001, **** < 0.0001.

	probability, *p*	intra-cluster time (s)	inter-cluster time (min)	duration of active phase (s)	video (h)
wild-type	0.57 ± 0.04	21.93 ± 2.05	29.07 ± 2.99	29.45 ± 4.29	60
*nlp-49*(−)	0.71 ± 0.03	16.67 ± 1.14	29.96 ± 2.88	39.97 ± 4.93	54
*seb-3*(−)	0.62 ± 0.03	23.45 ± 2.06	28.12 ± 2.92	37.06 ± 5.28	60
**Statistical analysis: wild-type versus *nlp-49*(−)**					
*p*-value	<0.0001	<0.0001	0.5185	0.0002	
significance	****	****	n.s.	***	
**Statistical analysis: wild-type versus *seb-3*(−)**					
*p*-value	0.0025	0.1157	0.4826	0.0023	
significance	**	n.s.	n.s.	**	

#### (ii) Tap arousal experiments

Sixteen hours before the experiment, late L4 animals were picked onto plates seeded with 90 µl of OP50 overnight culture and left to dry at room temperature for 14–16 h. Three or four plates of 50–100 animals per plate were tested per trial, with each genotype tested in at least three trials. Multi-worm tracking was performed using a Dinolite camera (AM4113ZT) positioned above the plates, which recorded videos at 5 frames per second. An additional white LED backlight was used to improve contrast. Video recording was started 2 min after the plate was placed on the stage. Animals were recorded for 20 s before taps were applied and then for a further 5 min. Taps were applied manually to the bottom of the assay plate. Tracks were analysed using matlab (MathWorks) with the Multi-Worm Tracker [23]. Area under the curve (AUC) measurements were performed using GraphPad Prism, with baseline values used for each genotype being the speed measurements recorded before the stimulus was applied. AUC was determined for 10–24 s post-stimulus to exclude the speed of the initial reversal [24].

#### (iii) Unsupervised behavioural tracking experiments

Recordings and analysis were performed as described in [19,20] using WormTracker 2.0 software. Briefly, tracking was carried out on 3 cm low peptone plates (standard NGM, with modifications: agar increased to 2%, peptone decreased to 0.013%). Low peptone plates were dried for 24 h at room temperature before use. On the day of tracking, plates were seeded with 20 µl of OP50 overnight culture and allowed to dry. Worms were moved to their tracking plate using a mounted eyelash hair and allowed to acclimatize for 30 min. Mutant/transgenic animals were tracked together with N2 controls. At least 17 animals were analysed per genotype. Recording for each strain was randomized across multiple trackers and across multiple days. Clustering analysis was performed by first analysing mutant videos with Tierpsy Tracker [25] to extract behavioural features for each worm which are averaged to create a single feature vector for each strain. Average feature vectors were then *z*-normalized across all the strains shown in figure 2*a,c*. Heat maps were produced by hierarchical clustering using complete linkage and Euclidean distance. To compare the phenotypic difference between *seb-3*(*tm1848*) and *nlp-49*(*gk546875*) with the difference observed in a large set of mutants, we used the same features as in figure 2*a,c,* but the *z*-normalization was performed across all of the strains in [19] and the two new mutants before calculating pairwise Euclidean distances. To identify features that showed significant differences between N2 and *seb-3*(*tm1848*) or *nlp-49*(*gk546875*) mutants, we performed multiple *t*-tests, controlling for false discovery rate (5%) using the Benjamini–Krieger–Yekutieli method. To compare the same feature between all genotypes tested, we performed a one-way ANOVA with Holm–Sidak's post-test for multiple comparisons.

*Statistical analysis* for all experiments was performed using GraphPad Prism v. 6.0. Exact statistical tests used are provided in the figure legends.

### (c) Receptor activation assays

Assays were performed as described [26]. Briefly, *seb-3/C18B12.2* cDNA was cloned into the pcDNA3.1(+) TOPO expression vector (Thermo Fisher Scientific). A CHO-K1 cell line (PerkinElmer, ES-000-A24) stably expressing apo-aequorin (mtAEQ) targeted to the mitochondria as well as the human Gα16 was transiently transfected with the *seb*-*3* cDNA construct or the empty pcDNA3.1(+) vector (not shown) using Lipofectamine (Thermo Fisher Scientific). Cells were shifted to 28°C 1 day later, and collected 2 days post-transfection in BSA medium (DMEM/Ham's F12 with 15 mM HEPES, without phenol red, 0.1% BSA) loaded with 5 µM coelenterazine h (Thermo Fisher Scientific) for 4 h. A total of 25 000 cells per well were exposed to synthetic peptides in BSA medium, and aequorin bioluminescence was recorded on a MicroBeta LumiJet luminometer (PerkinElmer, Waltham, MA) for 30 s in quadruplicate. After 30 s of ligand-stimulated calcium measurements, Triton X-100 (0.1%) was added to obtain a measure of the maximum cell Ca^2+^ response. BSA medium without peptides was used as a negative control and 1 µM ATP was used to check the functional response of the cells. Half-maximal effective concentration (EC_50_) values were calculated from dose–response curves, constructed using a nonlinear regression analysis, with the sigmoidal dose–response equation (Prism v. 6.0).

### (d) Confocal microscopy

Images were acquired using a Zeiss LSM 710 or 780 and *z*-stacks generated using Fiji (ImageJ). Animals were immobilized in 75 mM sodium azide on agarose pads (2% in M9 buffer (22 mM KH_2_PO_4_, 42 mM Na_2_HPO_4_, 86 mM NaCl)).

## 3. Results

**Figure 1.**
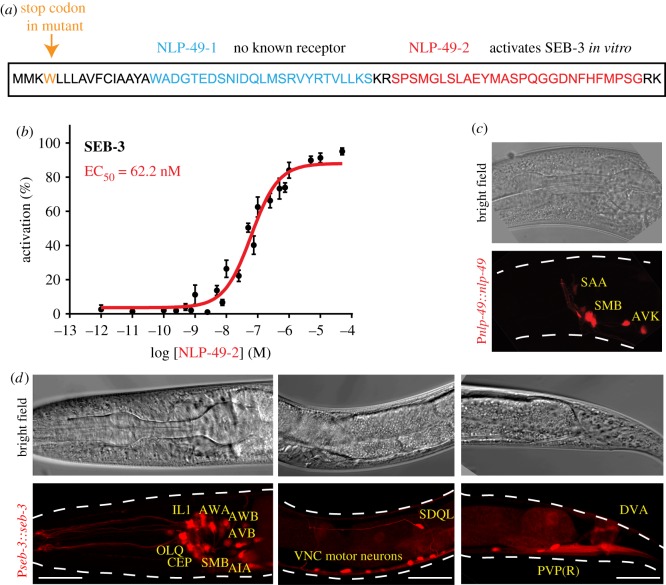
*nlp-49* and *seb-3* are expressed in the nervous system. (*a*) The peptide precursor encoded by *nlp-49* contains two peptide sequences, designated NLP-49-1 (blue) and NLP-49-2 (red). The mutant allele *gk546875* used in this study is a G→A missense mutation resulting in an amino acid change from W to a stop codon at position 4, resulting in the loss of expression of both peptides (https://wormbase.org). (*b*) Dose response curve for activation of SEB-3 by NLP-49-2 peptides (EC_50_ = 62.2 nM, *n* ≥ 9). (*c*) Representative micrograph showing P*nlp-49*::*nlp-49*::*SL2-mKate2* expression in SMB and AVK neurons, and putatively in the SAA neurons. (*d*) Micrographs of the head, midbody and tail region of animals expressing P*seb-3*::*seb-3*::*SL2-mKate2*, showing the broad nervous system expression of this transgene. VNC, ventral nerve cord. Some of the neurons identified in this study are indicated; for a full list see electronic supplementary material, table S2. Scale bar, 20 µm.

The *C. elegans* GPCR SEB-3 has been implicated in the regulation of different behavioural states, such as roaming behaviour, the control of sensory responsiveness during lethargus, and male mating behaviour [27,28]. However, the identity of ligands for this receptor remains to be discovered. To further characterize the neuromodulatory functions of SEB-3, we set out to identify its ligand(s) using a reverse pharmacology approach. We expressed SEB-3 in Chinese hamster ovary (CHO) cells together with the calcium indicator aequorin and the promiscuous Gα_16_ protein. We then challenged these cells with a synthetic library of over 300 isolated and predicted *C. elegans* peptides and monitored receptor activation via the calcium-based aequorin read-out. A single peptide encoded by the NLP-49 precursor (NLP-49-2, SPSMGLSLAEYMASPQGGDNFHFMPSamide) activated SEB-3 in this screen (figure 1*a*). We next tested a concentration series to determine the ligand's potency. NLP-49-2 dose-dependently activated SEB-3 with a half-maximal effective concentration (EC_50_) of 62 nM (figure 1*b*), within the expected nanomolar range of neuropeptide–receptor interactions, but did not evoke a calcium response in cells transfected with an empty vector (not shown). The precursor gene *nlp-49* (*H05L03.3*), previously shown to be involved in regulating fat storage [29], encodes two peptides, NLP-49-1 and NLP-49-2 (figure 1*a*), both of which have been identified by mass spectrometry in *C. elegans* [11]. To date, no receptor is known to be activated by NLP-49-1. The putative receptor for NLP-49-2, SEB-3, is a member of a largely uncharacterized family of secretin receptors of bilaterian origin, which is evolutionarily conserved from protostomes to cephalochordates, but appears to have been lost in vertebrates [5,30]. So far, SEB-3 is the only member of this family for which a ligand has been identified. Previous reports suggested that SEB-3 is similar to mammalian CRF receptors [16,27,28]. Recent phylogenetic evidence indicates that SEB-3 is not a direct orthologue of these receptors, but is most closely related to pigment-dispersing factor (PDF) receptors [5,30].

To further characterize the role of NLP-49 *in vivo*, we first determined the expression pattern of this neuropeptide gene using a reporter line expressing the full-length genomic DNA of *nlp-49* and a 2 kb promoter region (P*nlp-49*::*nlp-49 gDNA*::*SL2-mKate2*). Expression of this reporter was restricted to a few head neurons, which we identified as the SMB and AVK neurons. We also observed fainter expression in neurons that are anatomically consistent with the SAA neurons (figure 1*c*). In addition, consistent with a previous report [28], we found that a transgenic reporter construct for *seb-3*, using a 2 kb upstream region together with full intronic sequences, shows broad expression in the nervous system (figure 1*d*; see electronic supplementary material, table S2 for a full list of *seb-3-*expressing cells identified in this study).

An *nlp-49* allele obtained from the Million Mutation Project [31] results in an early stop codon at position 4, leading to the loss of both NLP-49 peptides (figure 1*a*). To investigate whether NLP-49 peptides interact with SEB-3 *in vivo*, we first investigated the locomotion phenotypes of mutants lacking SEB-3 or NLP-49 peptides. We used an automated tracking system to record videos of freely moving wild-type, mutant and transgenic rescue animals and measure a large number of features related to body shape and movement dynamics [19,20]. A large-scale survey of uncoordinated mutants using the WormTracker previously indicated that these measurements provide a useful behavioural fingerprint for classifying phenotypic similarity and revealing potential functional relationships; for example, loss-of-function alleles of the same gene (e.g. *unc-2, unc-4*), or mutants affecting components of known complexes (e.g. *unc-7/unc-9*, *unc-38/unc-63*), generally showed very similar phenotypic profiles [19].

**Figure 2.**
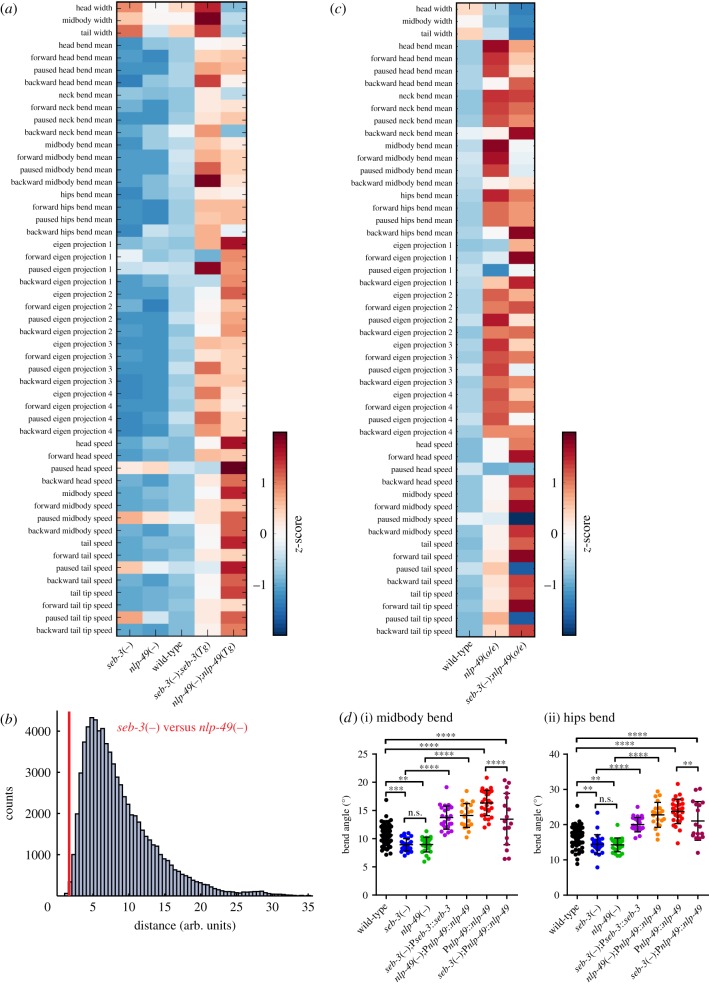
*nlp-49* and *seb-3* regulate locomotion. *seb-3*(*tm1848*) is referred to as *seb-3*(−) and *nlp-49*(*gk546875*) as *nlp-49*(−). (*a*) Heat map comparing behavioural phenotypes of wild-type, *seb-3*(−), *nlp-49*(−), *seb-3*(−);*seb-3*::*seb-3* (*seb-3* Tg), and *nlp-49*(−);*nlp-49*::*nlp-49* (*nlp-49* Tg). (*b*) Pairwise Euclidian distances (after *z*-normalization) for *nlp-49*(−) and *seb-3*(−) mutants compared with all previously tracked strains [19,20]. The *y*-axis indicates strain count. The comparison for *seb-3* versus *nlp-49* is shown in red. (*c*) Heat map comparing behavioural phenotypes of wild-type, *nlp-49*(*o/e*) and *seb-3*(−);*nlp-49*(*o/e*). For heat maps, the colours show the *z*-score of each feature calculated across all strains. (*d*) To identify features significantly different between wild-type and each single mutant, multiple *t*-tests were performed, controlling for false discovery rate (5%). Body bend features are compared between all genotypes tested. (i) Midbody bend angle, (ii) hips bend angle. Error bars indicate mean ± s.d. Statistical tests: one-way ANOVA, Holm–Sidak's post-test. *p*-values indicated by n.s., not significant, ** < 0.01, *** < 0.001 and **** < 0.0001. Raw data for all parameters are shown in electronic supplementary material, table S3. See also figure S1.

We first tracked outcrossed strains carrying loss-of-function mutations in *seb-3* and *nlp-49*. We observed that both *seb-3*(*tm1848*) and *nlp-49*(*gk546875*) mutants showed remarkably similar locomotion phenotypes. When compared with wild-type, the s*eb-3* and *nlp-49* strains showed similar changes for most locomotion features; by contrast, statistical analysis did not reveal any significant differences between *seb-3*(*tm1848*) and *nlp-49*(*gk546875*) mutants (multiple *t*-tests, Benjamini–Krieger–Yekutieli method, false discovery rate (*Q*) = 5%) (see electronic supplementary material, table S3 for raw values). Figure 2*a* shows a heat map of normalized differences calculated between wild-type and each mutant. To assess whether these phenotypes were due to *seb-3* and *nlp-49*, we also tracked *seb-3*(*tm1848*) and *nlp-49*(*gk546875*) animals rescued by transgenically re-expressing each gene under its own promoter, and compared these strains' behaviour with the wild-type. As transgenic lines were generated by microinjection, they are maintained as extrachromosomal arrays and are high-copy [32]. Therefore, we expected these transgenes to cause overexpression of SEB-3/NLP-49. Overexpression of neuropeptide precursor genes or neuropeptide receptors has previously been shown to cause gain-of-function phenotypes opposite to those seen in the respective null mutants (see, for example, [33–36]). Our data indicated that both neuropeptide and receptor rescue strains showed similar locomotion changes compared with wild-type, and that these changes were generally in the opposite direction to those seen for *nlp-49* and *seb-3* mutants (figure 2*a*). We then tested, using hierarchical clustering analysis, if locomotion profiles of *nlp-49* and *seb-3* mutants would cluster with each other more closely than with other strains. Indeed, we found that when computing pairwise distances between *nlp-49*(*gk546875*) and *seb-3*(*tm1848*) and all mutants in our database, *nlp-49* and *seb-3* mutants clustered more closely together than with previously tracked mutants [19] (figure 2*b*). These findings are consistent with NLP-49 and SEB-3 acting in the same molecular pathway to modulate locomotion. To identify features that are significantly different between wild-type and *seb-3* or *nlp-49* mutants, we performed multiple *t*-tests for all features between wild-type and each mutant (controlling for *Q* = 5%). Significant effects were observed in features measuring body curvature, i.e. mid-body bend, hips bend, eigen projection 2 and eigen projection 3 (figure 2*d*; electronic supplementary material, figure S1B).

We next crossed *nlp-49*(*o/e*) animals with *seb-3* deletion mutants to determine if loss of SEB-3 would affect locomotion changes resulting from overexpression of NLP-49 peptides. Interestingly, we found that effects on body curvature, such as some body bend angle features and eigen projection features, are generally attenuated in *nlp-49* overexpressing animals lacking SEB-3 (*seb-3*(−);*nlp-49*(*o/e*)). However, there are several features that do not show a trend towards a more wild-type profile, or are unchanged (figure 2*c*). These phenotypes could result from NLP-49-2 effects independent of SEB-3 or increased levels of NLP-49-1 peptides, which do not activate SEB-3. Figure 2*d*(i,ii) shows examples of specific features comparing differences in body curvature among the strains tested. Both *seb-3*(*tm1848*) and *nlp-49*(*gk546875*) show significantly smaller body bend angles compared with wild-type (see electronic supplementary material, figure S1A for representative traces; also electronic supplementary material, figure S1B), and transgenic rescue for the expression of these genes leads to an increased bend angle compared with the respective mutant strains. In addition, *nlp-49*(*o/e*) animals show generally increased bend angles compared with wild-type, and loss of SEB-3 reduces these effects.

**Figure 3.**
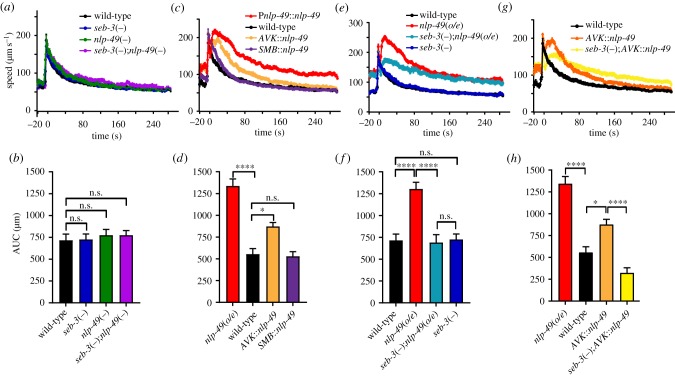
*nlp-49* and *seb-3* promote locomotor arousal. *seb-3*(*tm1848*) is referred to as *seb-3*(−) and *nlp-49(gk546875)* as *nlp-49*(−). Tap stimulation is provided at *t* = 0. (*a*) Speed traces of *nlp-49*(−) and *seb-3*(−) single mutants, and *seb-3*(−);*nlp-49*(−) double mutants, together with wild-type controls following tap. (*b*) Quantification of locomotor arousal responses for (*a*) showing the area under the curve for 10–24 s post-tap. (*c*) Speed traces of transgenic animals overexpressing *nlp-49* using its own promoter (*nlp-49*(*o/e*)), or specifically in the SMB (using the *flp-12* promoter) [38] or AVK (using the *flp-1* promoter) [39] neurons following tap. (*d*) Quantification of arousal responses for (*c*). (*e*) Speed traces of animals overexpressing *nlp-49* from its own promoter, with or without the mutant allele for *seb-3* (*seb-3*(−)), together with wild-type and *seb-3*(−) controls following tap. (*f*) Quantification of arousal responses in (*e*). (*g*) Speed traces of animals overexpressing *nlp-49* from the AVK neurons, with or without the mutant allele for *seb-3*, together with wild-type controls following tap. (*h*) Quantification of arousal responses in (*g*). *nlp-49*(*o/e*) is shown for comparison. *n* > 200 for all genotypes. Error bars indicate mean ± s.e.m. Statistical tests: one-way ANOVA, Sidak's post-test. *p*-values indicated by n.s., not significant, * < 0.05 and **** < 0.0001.

We next tested if SEB-3 and NLP-49 regulate another aspect of locomotion: touch-evoked arousal. When animals are exposed to a non-localized mechanosensory stimulus, they undergo a transient increase in locomotor activity that persists for 1–2 min [37]. To determine whether this behaviour is affected by *nlp-49* and *seb-3*, five taps were rapidly delivered to the Petri dish housing the animals and the speed of these animals was recorded using automated tracking software [23]. To quantify this locomotor hyperactivity (arousal) following mechanosensory stimulation, we used the average area underneath the curve (AUC) for each trace for 10–24 s post-stimulus. We did not include the first 10 s post-tap as the acute effects of tap (less than 10 s) are largely due to the initial escape response comprising backward locomotion [24]. Deletion mutants of *seb-3* and *nlp-49*, as well as *seb-3*;*nlp-49* double mutants, showed essentially wild-type behaviour (figure 3*a,b*); however, overexpression of *nlp-49* led to a robust increase in arousal following the tap stimulus compared with wild-type (figure 3*c,d*). Even taking into account the basal increase in locomotion speed observed for *nlp-49*(*o/e*) animals, we observed a significantly greater increase in motor activity when we compared the speed observed after tap with the baseline speed recorded before tap. The increased locomotor hyperactivity seen in *nlp-49*(*o/e*) therefore seems to be due to the increase in speed post-stimulus being longer lasting than in wild-type controls.

We then tested if expression of *nlp-49* from particular head neurons was specifically required for this hyper-aroused phenotype. Surprisingly, expression of *nlp-49* from the AVK interneurons, but not from the SMB motor neurons, led to increased locomotor arousal (figure 3*c,d*), suggesting that specific inputs to AVK are required for arousal. We next asked if loss of SEB-3 would affect the hyper-arousal resulting from elevated NLP-49 peptides. Indeed, we found that loss of SEB-3 strongly attenuated the arousal effect of *nlp-49* overexpression (figure 3*e,f*). Interestingly, although loss of SEB-3 resulted in reduced locomotor arousal after tap, it did not affect the higher baseline speed of *nlp-49*(*o/e*) transgenic animals (figure 3*e*). The basal increase in locomotion speed observed in *nlp-49* overexpressors could therefore result from SEB-3-independent effects or increased levels of NLP-49-1 peptides. We also found that the effects of expressing *nlp-49* from AVK were dependent on SEB-3, as *AVK*::*nlp-49* transgenic animals containing a *seb-3*(*tm1848*) mutation showed significantly reduced locomotor arousal compared with *AVK*::*nlp-49* animals containing intact SEB-3 (figure 3*g,h*). These data indicate that NLP-49 and SEB-3 act together to affect locomotion in response to aversive stimuli in addition to the changes observed in unstimulated conditions.

**Figure 4.**
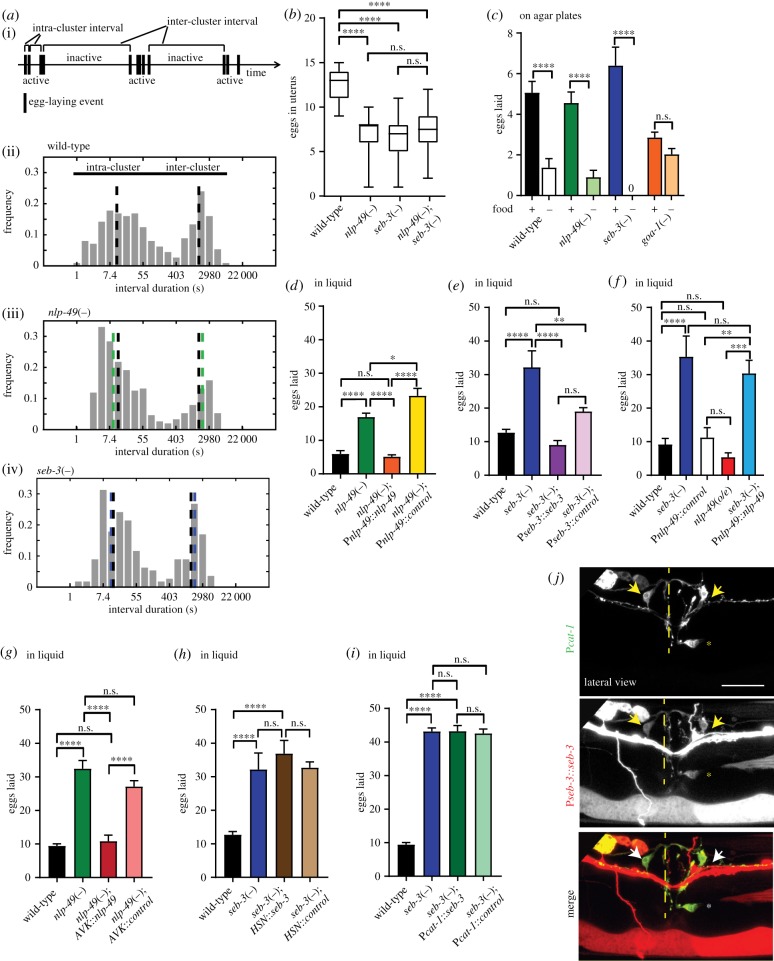
(*Overleaf*.) *nlp-49* and *seb-3* regulate egg-laying behaviour. *seb-3*(*tm1848*) is referred to as *seb-3*(−) and *nlp-49*(*gk546875*) as *nlp-49*(−). (*a*)(i) Representative temporal pattern for egg-laying showing active phases during which eggs are laid, interspersed by longer inactive phases. The time between egg-laying events during the active phase is the *inter-cluster time*, and the time between active phases is the *intra-cluster time*; (ii) histogram of interval times between egg-laying events for wild-type animals, with the interval duration in log scale. The location of the two peaks indicated by black dashed lines corresponds to the intra-cluster and inter-cluster time constants, estimated using maximum-likelihood analysis [44]; (iii) interval times between egg-laying events for *nlp-49*(−) animals; (iv) interval times between egg-laying events for *seb-3*(−) animals. Intra- and inter-cluster time constants for mutants are indicated in green (*nlp-49*(−)) or blue (*seb-3*(−)), with wild-type shown in black for comparison. See also table 1. (*b*) Eggs retained in the uterus for wild-type, *nlp-49*(−), *seb-3*(−) and *seb-3*(−);*nlp-49*(−) animals*,* at 16 h post-L4. 3 replicates, *n* > 15 per replicate. The box-and-whisker plot shows the box extending from the 25th to the 75th percentile, the line in the middle indicating the median. Whiskers extend from minimum to maximum. (*c*) Eggs laid on agar plates in the presence or absence of food (indicated by + or − beneath the graph) for wild-type, *nlp-49*(−) and *seb-3*(−) mutant animals, with *goa-1* mutants as a positive control. *n* = 3 replicates, *n* = 12 per condition per replicate. Eggs laid in liquid (average for 10 worms) for (*d*) wild-type, *nlp-49*(−), and *nlp-49* mutants expressing a rescue transgene for *nlp-49* expression under its own promoter or a control *mKate2* transgene; (*e*) wild-type, *seb-3*(−), and *seb-3* mutants expressing a rescue transgene for *seb-3* expression under its own promoter or a control *mKate2* transgene; (*f*) wild-type, *seb-3* mutants, animals overexpressing *nlp-49* under its own promoter or a control *mKate2* transgene, and animals overexpressing *nlp-49* in a *seb-3*(−) background; (*g*) wild-type, *nlp-49*(−), and *nlp-49* mutants containing a transgene for *nlp-49* expression in AVK (P*flp-1*) or a control *mKate2* transgene; (*h*) wild-type, *seb-3*(−), and *seb-3* mutants expressing *seb-3* in HSN (P*egl-6*) or a control *mKate2* transgene; (*i*) wild-type, *seb-3*(−), and *seb-3* mutants expressing *seb-3* using a *cat-1* promoter or a control *gfp* transgene. (*j*) Micrographs showing co-localization of P*seb-3*::*seb-3*::*SL2-mKate2* with *cat-1-*positive cells. The position of the vulval slit is indicated by the dashed line. HSN is shown by the asterisk and VC4/VC5 are indicated by arrows. Scale bar, 20 µm. For egg-laying assays in liquid (*d–i*), eggs were counted after 60 min, error bars indicate mean ± s.e.m., *n* > 3 replicates, *n* = 60 per genotype. Statistical tests: one-way ANOVA, Sidak's post-test. *p*-values indicated by n.s., not significant, * < 0.05, ** < 0.01, *** < 0.001 and **** < 0.0001.

Changes in locomotion and egg-laying events appear to be coordinated [40,41] and some neuropeptides that regulate locomotion also affect egg-laying [36,42,43]. Detailed study revealed that egg-laying events tend to be clustered, with events within clusters (active phases) usually separated by approximately 20 s (intra-cluster interval). These clusters take place between long inactive periods of 20–30 min (inter-cluster interval) (figure 4*a*(i,ii)). The time constants for these intervals, and the probability of egg-laying events occurring during each cluster (probability, *p*), can be inferred by obtaining maximum-likelihood estimates of the inter- and intra-cluster time constants from egg-laying interval data [21,44,45]. We found that *nlp-49* and *seb-3* deletion mutants show egg-laying constitutive behaviours, as indicated by a significantly higher probability of egg-laying during the active phase and longer active phase duration (figure 4*a*(iii,iv); table 1). Inactive state durations are not significantly affected (figure 4*a* and table 1). In addition, *nlp-49*(*gk546875*) animals showed on average a shorter duration between egg-laying events during the active phase (figure 4*a*(iii) and table 1). Representative raster plots for these mutants are shown in electronic supplementary material, figure S2A. Consistent with a higher rate of egg-laying during the reproductive phase, single deletion mutants for both genes, as well as the double mutant, also showed significantly fewer eggs retained in the uterus as day 1 adults compared with wild-type (figure 4*b*). Double *nlp-49;seb-3* mutants did not show an additive effect compared with single mutants, consistent with these genes acting in the same genetic pathway to modulate egg-laying (figure 4*b*). Conversely, overexpression of *nlp-49* showed more eggs retained in the uterus (electronic supplementary material, figure S2B). Thus, *nlp-49* and *seb-3* loss-of-function mutants both showed detectable egg-laying constitutive phenotypes.

We next investigated how NLP-49 and SEB-3 affect the modulation of egg-laying by external stimuli, such as in the presence or absence of food, or culture on liquid or solid medium. We found that neither *nlp-49*(*gk546875*) nor *seb-3*(*tm1848*) animals laid eggs in the absence of food, unlike egg-laying hyperactive mutants such as *goa-1*(*n1134*) (figure 4*c*). However, mutants of both genes showed increased egg-laying in liquid medium compared with wild-type controls (figure 4*d,e*). Additionally, *nlp-49;seb-3* double mutants did not show significantly more egg-laying than either single mutant (electronic supplementary material, figure S2C). We could rescue the mutant phenotype by transgenic re-expression of *nlp-49* (figure 4*d*) and *seb-3* (figure 4*e*). However, the case for *seb-3* is complicated by the fact that a control line expressing *mKate2* using the *seb-3* promoter in a *seb-3*(*tm1848*) background, expected to behave similarly to animals carrying *seb-3*(*tm1848*) alone, showed no statistically significant difference in behaviour compared with the *seb-3*(*tm1848*);P*seb-3*::*seb-3* line (*p*-value 0.09; figure 4*e*). This suggests that transgene expression using the *seb-3* promoter affects egg-laying, making it difficult to conclude for certain if the rescuing effect of P*seb-3*::*seb-3* is due to re-expression of *seb-3* or due to promoter/transgene effects. We next tested if an interaction could be observed between NLP-49 and SEB-3 by examining egg-laying behaviour in an *nlp-49* over-expressing line. Although there was a trend towards a lower egg-laying rate in *nlp-49*(*o/e*) animals (P*nlp-49*::*nlp-49* in a wild-type background) compared with a control line expressing *mKate2* using the *nlp-49* promoter, this difference did not reach statistical significance. However, we also found that *seb-3*(*tm1848*);P*nlp-49*::*nlp-49* animals showed a similar phenotype to animals carrying the *seb-3*(*tm1848*) allele alone, which is consistent with NLP-49 and SEB-3 acting in the same pathway (figure 4*f*). As FLP-1 peptides released from AVK also modulate egg-laying behaviour [22], we next tested if AVK expression of *nlp-49* (using the *flp-1* promoter) could rescue the mutant phenotype. Indeed, we found that transgenic expression of *nlp-49* from the AVK neurons in an *nlp-49*(−) background showed significant rescue compared with controls (figure 4*g*). Lastly, we tested if SEB-3 acts directly in egg-laying motor neurons to modulate egg-laying behaviour. SEB-3 is expressed in the HSN and VC4/5 motor neurons, consistent with this possibility (figure 4*j*). However, when we expressed SEB-3 in HSN using an *egl-6* promoter [46], or using a *cat-1* promoter, which drives expression in VC4/5 and HSN in addition to other dopaminergic and serotonergic neurons [47], in a *seb-3*(*tm1848*) mutant background, we did not observe rescue of the *seb-3* mutant phenotype (figure 4*h,i*), suggesting that SEB-3 expression in the egg-laying circuit is not sufficient for its effect on egg-laying. Thus, SEB-3 may function in neurons apart from VC4/5 or the HSNs, or these neurons in combination with others, to modulate egg-laying behaviour.

## 4. Discussion

Here, we showed for the first time, to our knowledge, a biochemical interaction between the SEB-3 receptor and NLP-49 peptides. We also demonstrated that NLP-49/SEB-3 signalling regulates multiple behaviours in *C. elegans*. Unlike *seb-3*, which is broadly expressed, *nlp-49* appears to be expressed only in a small number of head neurons: AVK, SMB and putatively the SAA neurons, which interestingly are all connected to one another via chemical synapses or gap junctions [8]. It is therefore possible that activation of one of these neurons can synchronize peptide release from other *nlp-49-*expressing cells. Our findings suggest that neuromodulator release from only a few neurons can affect diverse behaviours by potentially acting through a receptor that is widely present in the nervous system. This form of signalling provides another layer of communication in addition to existing chemical synapse/gap junction connections, allowing signals to be transmitted over greater anatomical distances and longer timescales [48]. Multiple neuromodulator signals can also be coordinated to consolidate important behaviours or to provide a means by which the animal can fine-tune its responses to sensory cues [6,7]. In the case of arousal (and egg-laying), expression of *nlp-49* in AVK appears to be the most important to drive this behaviour. AVK has not previously been implicated in the control of locomotion; indeed a different neuropeptide that controls arousal-like behaviour, PDF-1, appears to act primarily in a different set of neurons, AIY and ASK [49,50]. Interestingly, we have found that the receptor (FRPR-3) for another neuropeptide (FLP-20) required for arousal in response to mechanosensory signals is also expressed in AVK [37], suggesting that multiple neuropeptide systems may function together to promote arousal.

We found that NLP-49/SEB-3 signalling also modulates egg-laying behaviour. Egg-laying is regulated by external conditions, such that stressful environments including thermal stress, hypertonic salt solutions and starvation tend to suppress this behaviour [22,51–54]. *nlp-49* and *seb-3* deletion mutants both show an increased rate of egg-laying not only under permissive conditions, but also in a stressful environment (in liquid). Surprisingly, although SEB-3 is expressed in egg-laying motor neurons, receptor expression in these neurons was not sufficient to rescue the egg-laying phenotype, suggesting SEB-3 may function outside the egg-laying circuit to control egg-laying behaviour. Additionally, overexpression of *nlp-49* promotes locomotor hyperactivity via SEB-3 in response to an aversive tap stimulus. Together, our data suggest that NLP-49/SEB-3 signalling may be stress-responsive, such that decreased signalling is indicative of a low-stress state, and increased signalling correlates with a high-stress state. The CRF peptide system in mammals is thought to mediate stress and anxiety responses (see, for example, [55,56]). Although the CRF system appears to have been lost in nematodes [5,30,57], SEB-3 belongs to the same secretin superfamily of receptors as mammalian CRF receptors, as well as invertebrate PDF receptors. PDF peptides were previously shown to also regulate arousal responses in *C. elegans* [50,58]. NLP-49/SEB-3 may therefore be another member of the secretin family of GPCRs, which modulates behaviour in stress conditions.

Overall, the behavioural phenotypes of *nlp-49* and *seb-3* loss-of-function mutants are remarkably similar. In particular, *nlp-49* and *seb-3* mutants differ from wild-type in many of the same locomotor features, and the *nlp-49* and *seb-3* rescue lines likewise show similar behavioural fingerprints with respect to locomotion. Indeed, the locomotor phenotypes of *nlp-49* and *seb-3* mutants cluster closer than expected relative to a large dataset of strains. Thus, our behavioural analysis supports the *in vitro* data indicating that NLP-49-2 peptides are ligands for the SEB-3 receptor, and demonstrates the utility of high-content behavioural phenotyping to validate ligand–receptor pairs identified through de-orphanization studies.

The *C. elegans* genome encodes a diverse array of neuropeptides [11]. Expression of FLP, NLP and *INS* peptides is widespread in the nervous system [1,9,39,59], and these peptides have been implicated in a wide variety of behaviours [1,60]. Many *C. elegans* neuropeptides and their receptors also have orthologues in other animals, including humans [30,57]. SEB-3 is, we believe, the first identified de-orphaned receptor of a bilaterian family of secretin PDF-like neuropeptide receptors that evolved before the split of proto- and deuterostomian animals [5]. Members of this ancient neuropeptide receptor family are evolutionarily conserved in molluscs, annelids and deuterostomian invertebrates [5]. It will be interesting to see if neuropeptides similar to *C. elegans* NLP-49 activate SEB-3 orthologues in these species. Furthermore, preliminary investigations on the network properties of neuropeptide signalling in the (relatively few) examples where peptide–receptor pairs have been identified in *C. elegans* suggest that this form of communication takes place largely beyond physical connections, i.e. in a more ‘wireless’ manner [48]. Large-scale de-orphanization screens (for example [61]), combined with *in vivo* phenotyping of receptor and peptide mutants, provide a potential avenue to build a more complete connectome of neuropeptide signalling. This multilayer connectome will prove useful as a model to understand larger nervous systems, such as the human brain.

## Supplementary Material

Supplementary information

## Supplementary Material

Table S3 Tracking Data

## Data accessibility

Additional data have been deposited as supplementary material. Raw video files for *seb-3* and *nlp-49* mutants and transgenic lines are available from the authors by request.

## References

[1] Li C, Kim K 2008 Neuropeptides. *WormBook*. See http://www.wormbook.org/chapters/www_neuropeptides/neuropeptides.html. 10.1895/wormbook.1.142.1 PMC2749236 18819171

[2] Hartenstein V 2006 The neuroendocrine system of invertebrates: a developmental and evolutionary perspective. J. Endocrinol. 190, 555–570. (10.1677/joe.1.06964) 17003257

[3] Quillet R, Ayachi S, Bihel F, Elhabazi K, Ilien B, Simonin F 2016 RF-amide neuropeptides and their receptors in Mammals: pharmacological properties, drug development and main physiological functions. Pharmacol. Ther. 160, 84–132. (10.1016/j.pharmthera.2016.02.005) 26896564

[4] Heinrichs M, Domes G 2008 Neuropeptides and social behaviour: effects of oxytocin and vasopressin in humans. Prog. Brain Res. 170, 337–350. (10.1016/S0079-6123(08)00428-7) 18655894

[5] Elphick MR, Mirabeau O, Larhammar D 2018 Evolution of neuropeptide signalling systems. J. Exp. Biol. 221, jeb151092 (10.1242/jeb.151092) 29440283 PMC5818035

[6] Bargmann CI 2012 Beyond the connectome: how neuromodulators shape neural circuits. Bioessays 34, 458–465. (10.1002/bies.201100185) 22396302

[7] Marder E 2012 Neuromodulation of neuronal circuits: back to the future. Neuron 76, 1–11. (10.1016/j.neuron.2012.09.010) 23040802 PMC3482119

[8] White JG, Southgate E, Thomson JN, Brenner S 1986 The structure of the nervous system of the nematode *Caenorhabditis elegans*. Phil. Trans. R. Soc. Lond. B 314, 1–340. (10.1098/rstb.1986.0056) 22462104

[9] Li C, Nelson LS, Kim K, Nathoo A, Hart AC 1999 Neuropeptide gene families in the nematode *Caenorhabditis elegans*. Ann. N. Y. Acad. Sci. 897, 239–252. (10.1111/j.1749-6632.1999.tb07895.x) 10676452

[10] Hobert O 2013 The neuronal genome of *Caenorhabditis elegans*. WormBook. See http://www.wormbook.org/chapters/www_neuronalgenome/neuronalgenome.html. 10.1895/wormbook.1.161.1 PMC4781646 24081909

[11] Van Bael S, Zels S, Boonen K, Beets I, Schoofs L, Temmerman L 2018 A *Caenorhabditis elegans* mass spectrometric resource for neuropeptidomics. J. Am. Soc. Mass Spectrom. 29, 879–889. 29299835 10.1007/s13361-017-1856-z

[12] Steiner DF 1998 The proprotein convertases. Curr. Opin. Chem. Biol. 2, 31–39. (10.1016/S1367-5931(98)80033-1) 9667917

[13] Jekely G, Melzer S, Beets I, Kadow ICG, Koene J, Haddad S, Holden-Dye L 2018 The long and the short of it–a perspective on peptidergic regulation of circuits and behaviour. J. Exp. Biol. 221, jeb166710 (10.1242/jeb.166710) 29439060

[14] Li C, Kim K 2014 Family of FLP peptides in *Caenorhabditis elegans* and related nematodes. Front. Endocrinol. 5, 150 (10.3389/fendo.2014.00150) PMC4196577 25352828

[15] Schioth HB, Fredriksson R 2005 The GRAFS classification system of G-protein coupled receptors in comparative perspective. Gen. Comp. Endocrinol. 142, 94–101. (10.1016/j.ygcen.2004.12.018) 15862553

[16] Cardoso JC, Pinto VC, Vieira FA, Clark MS, Power DM 2006 Evolution of secretin family GPCR members in the metazoa. BMC Evol. Biol. 6, 108 (10.1186/1471-2148-6-108) 17166275 PMC1764030

[17] Refojo D, Holsboer F 2009 CRH signaling molecular specificity for drug targeting in the CNS. Ann. N. Y. Acad. Sci. 1179, 106-119. (10.1111/j.1749-6632.2009.04983.x) 19906235

[18] Brenner S 1974 The genetics of *Caenorhabditis elegans*. Genetics 77, 71–94. 4366476 10.1093/genetics/77.1.71 PMC1213120

[19] Yemini E, Jucikas T, Grundy LJ, Brown AE, Schafer WR 2013 A database of *Caenorhabditis elegans* behavioral phenotypes. Nat. Meth. 10, 877–879. (10.1038/nmeth.2560) PMC3962822 23852451

[20] Brown AE, Yemini EI, Grundy LJ, Jucikas T, Schafer WR 2013 A dictionary of behavioral motifs reveals clusters of genes affecting *Caenorhabditis elegans* locomotion. Proc. Natl Acad. Sci. USA 110, 791–796. (10.1073/pnas.1211447110) 23267063 PMC3545781

[21] Branicky R, Miyazaki H, Strange K, Schafer WR 2014 The voltage-gated anion channels encoded by *clh-3* regulate egg laying in *C. elegans* by modulating motor neuron excitability. J. Neurosci. 34, 764–775. (10.1523/JNEUROSCI.3112-13.2014) 24431435 PMC3891957

[22] Waggoner LE, Hardaker LA, Golik S, Schafer WR 2000 Effect of a neuropeptide gene on behavioral states in *Caenorhabditis elegans* egg-laying. Genetics 154, 1181–1192. 10757762 10.1093/genetics/154.3.1181 PMC1460996

[23] Ramot D, Johnson BE, Berry TL Jr, Carnell L, Goodman MB The Parallel Worm Tracker: a platform for measuring average speed and drug-induced paralysis in nematodes. PLoS ONE 3, e2208 (10.1371/journal.pone.0002208) PMC2373883 18493300

[24] Rankin CH, Beck CD, Chiba CM 1990 *Caenorhabditis elegans*: a new model system for the study of learning and memory. Behav. Brain Res. 37, 89–92. (10.1016/0166-4328(90)90074-O) 2310497

[25] Javer A et al. 2018 An open source platform for analyzing and sharing worm behavior data. Nature Methods 15, 645–646. 30171234 10.1038/s41592-018-0112-1 PMC6284784

[26] Beets I, Janssen T, Meelkop E, Temmerman L, Suetens N, Rademakers S, Jansen G, Schoofs L 2012 Vasopressin/oxytocin-related signaling regulates gustatory associative learning in *C. elegans*. Science 338, 543–545. (10.1126/science.1226860) 23112336

[27] Jee C, Goncalves JF, LeBoeuf B, Garcia LR 2016 CRF-like receptor SEB-3 in sex-common interneurons potentiates stress handling and reproductive drive in *C. elegans*. Nat. Commun. 7, 11957 (10.1038/ncomms11957) 27321013 PMC4915151

[28] Jee C, Lee J, Lim JP, Parry D, Messing RO, McIntire SL 2013 SEB-3, a CRF receptor-like GPCR, regulates locomotor activity states, stress responses and ethanol tolerance in *Caenorhabditis elegans*. Genes Brain Behav. 12, 250–262. (10.1111/j.1601-183X.2012.00829.x) 22853648 PMC3848202

[29] Ashrafi K, Chang FY, Watts JL, Fraser AG, Kamath RS, Ahringer J, Ruvkun G 2003 Genome-wide RNAi analysis of *Caenorhabditis elegans* fat regulatory genes. Nature 421, 268–272. (10.1038/nature01279) 12529643

[30] Mirabeau O, Joly JS 2013 Molecular evolution of peptidergic signaling systems in bilaterians. Proc. Natl Acad. Sci. USA 110, E2028–E2037. (10.1073/pnas.1219956110) 23671109 PMC3670399

[31] Thompson O et al. 2013 The million mutation project: a new approach to genetics in *Caenorhabditis elegans*. Genome Res. 23, 1749–1762. (10.1101/gr.157651.113) 23800452 PMC3787271

[32] Mello CC, Kramer JM, Stinchcomb D, Ambros V 1991 Efficient gene transfer in *C. elegans*: extrachromosomal maintenance and integration of transforming sequences. EMBO J. 10, 3959–3970. 1935914 10.1002/j.1460-2075.1991.tb04966.x PMC453137

[33] Bendena WG, Boudreau JR, Papanicolaou T, Maltby M, Tobe SS, Chin-Sang ID 2008 A *Caenorhabditis elegans* allatostatin/galanin-like receptor NPR-9 inhibits local search behavior in response to feeding cues. Proc. Natl Acad. Sci. USA 105, 1339–1342. (10.1073/pnas.0709492105) 18216257 PMC2234139

[34] Nelson MD, Janssen T, York N, Lee KH, Schoofs L, Raizen DM 2015 FRPR-4 Is a G-protein coupled neuropeptide receptor that regulates behavioral quiescence and posture in *Caenorhabditis elegans*. PLoS ONE 10, e0142938 (10.1371/journal.pone.0142938) 26571132 PMC4646455

[35] Nelson MD, Trojanowski NF, George-Raizen JB, Smith CJ, Yu C-C, Fang-Yen C, Raizen DM 2013 The neuropeptide NLP-22 regulates a sleep-like state in *Caenorhabditis elegans*. Nat. Commun. 4, 2846 (10.1038/ncomms3846) 24301180 PMC3867200

[36] Chang YJ, Burton T, Ha L, Huang Z, Olajubelo A, Li C 2015 Modulation of locomotion and reproduction by FLP neuropeptides in the nematode *Caenorhabditis elegans*. PLoS ONE 10, e0135164 (10.1371/journal.pone.0135164) 26406995 PMC4583311

[37] Chew YL et al. 2018 An afferent neuropeptide system transmits mechanosensory signals triggering sensitization and arousal in *C. elegans*. Neuron. (10.1016/j.neuron.2018.08.003) PMC6162336 30146306

[38] Kim J et al. 2015 The evolutionarily conserved LIM homeodomain protein LIM-4/LHX6 specifies the terminal identity of a cholinergic and peptidergic *C. elegans* sensory/inter/motor neuron-type. PLoS Genet. 11, e1005480 (10.1371/journal.pgen.1005480) 26305787 PMC4549117

[39] Kim K, Li C 2004 Expression and regulation of an FMRFamide-related neuropeptide gene family in *Caenorhabditis elegans*. J. Comp. Neurol. 475, 540–550. (10.1002/cne.20189) 15236235

[40] Collins KM et al. 2016 Activity of the *C. elegans* egg-laying behavior circuit is controlled by competing activation and feedback inhibition. eLife 5, e21126 (10.7554/eLife.21126) 27849154 PMC5142809

[41] Hardaker LA, Singer E, Kerr R, Zhou G, Schafer WR 2001 Serotonin modulates locomotory behavior and coordinates egg-laying and movement in *Caenorhabditis elegans*. J. Neurobiol. 49, 303–313. (10.1002/neu.10014) 11745666

[42] Meelkop E, Temmerman L, Janssen T, Suetens N, Beets I, Van Rompay L, Shanmugam N, Husson SJ, Schoofs L 2012 PDF receptor signaling in *Caenorhabditis elegans* modulates locomotion and egg-laying. Mol. Cell. Endocrinol. 361, 232–240. (10.1016/j.mce.2012.05.001) 22579613

[43] Buntschuh I, Raps DA, Joseph I, Reid C, Chait A, Totanes R, Sawh M, Li C, Hart AC 2018 FLP-1 neuropeptides modulate sensory and motor circuits in the nematode *Caenorhabditis elegans*. PLoS ONE 13, e0189320 (10.1371/journal.pone.0189320) 29293515 PMC5749679

[44] Zhou GT, Schafer WR, Schafer RW 1998 A three-state biological point process model and its parameter estimation. IEEE Trans. Signal Process. 46, 2698–2707. (10.1109/78.720372)

[45] Waggoner LE, Zhou GT, Schafer RW, Schafer WR 1998 Control of alternative behavioral states by serotonin in *Caenorhabditis elegans*. Neuron 21, 203–214. (10.1016/S0896-6273(00)80527-9) 9697864

[46] Ringstad N, Horvitz HR 2008 FMRFamide neuropeptides and acetylcholine synergistically inhibit egg-laying by *C. elegans*. Nat. Neurosci. 11, 1168–1176. (10.1038/nn.2186) 18806786 PMC2963311

[47] Duerr JS, Frisby DL, Gaskin J, Duke A, Asermely K, Huddleston D, Eiden LE, Rand JB 1999 The *cat-1* gene of *Caenorhabditis elegans* encodes a vesicular monoamine transporter required for specific monoamine-dependent behaviors. J. Neurosci. 19, 72–84. (10.1523/JNEUROSCI.19-01-00072.1999) 9870940 PMC6782383

[48] Bentley B et al. 2016 The multilayer connectome of *Caenorhabditis elegans*. PLoS Comput. Biol. 12, e1005283 (10.1371/journal.pcbi.1005283) 27984591 PMC5215746

[49] Flavell SW, Pokala N, Macosko EZ, Albrecht DR, Larsch J, Bargmann CI 2013 Serotonin and the neuropeptide PDF initiate and extend opposing behavioral states in *C. elegans*. Cell 154, 1023–1035. (10.1016/j.cell.2013.08.001) 23972393 PMC3942133

[50] Choi S, Chatzigeorgiou M, Taylor KP, Schafer WR, Kaplan JM 2013 Analysis of NPR-1 reveals a circuit mechanism for behavioral quiescence in *C. elegans*. Neuron 78, 869–880. (10.1016/j.neuron.2013.04.002) 23764289 PMC3683153

[51] Schafer WR 2005 Egg-laying. *WormBook* (14 December 2005). See http://www.wormbook.org/chapters/www_egglaying/egglaying.html. 10.1895/wormbook.1.38.1 PMC4781431 18050396

[52] Schafer WR 2006 Genetics of egg-laying in worms. A. Rev. Genet. 40, 487–509. (10.1146/annurev.genet.40.110405.090527) 17094742

[53] Zhang M et al. 2008 A self-regulating feed-forward circuit controlling *C. elegans* egg-laying behavior. Curr. Biol. 18, 1445–1455. (10.1016/j.cub.2008.08.047) 18818084 PMC2621019

[54] McMullen PD, Aprison EZ, Winter PB, Amaral LA, Morimoto RI, Ruvinsky I 2012 Macro-level modeling of the response of *C. elegans* reproduction to chronic heat stress. PLoS Comput. Biol. 8, e1002338 (10.1371/journal.pcbi.1002338) 22291584 PMC3266876

[55] Reul JM, Holsboer F 2002 On the role of corticotropin-releasing hormone receptors in anxiety and depression. Dialogues Clin. Neurosci. 4, 31–46. 22033745 10.31887/DCNS.2002.4.1/jreul PMC3181666

[56] Risbrough VB, Stein MB 2006 Role of corticotropin releasing factor in anxiety disorders: a translational research perspective. Horm. Behav. 50, 550–561. (10.1016/j.yhbeh.2006.06.019) 16870185 PMC1884405

[57] Jekely G 2013 Global view of the evolution and diversity of metazoan neuropeptide signaling. Proc. Natl Acad. Sci. USA 110, 8702–8707. (10.1073/pnas.1221833110) 23637342 PMC3666674

[58] Chen D, Taylor KP, Hall Q, Kaplan JM 2016 The neuropeptides FLP-2 and PDF-1 act in concert to arouse *Caenorhabditis elegans* locomotion. Genetics. 204, 1151–1159. (10.1534/genetics.116.192898) 27585848 PMC5105848

[59] Pierce SB et al. 2001 Regulation of DAF-2 receptor signaling by human insulin and *ins-1*, a member of the unusually large and diverse *C. elegans* insulin gene family. Genes Dev. 15, 672–686. (10.1101/gad.867301) 11274053 PMC312654

[60] Frooninckx L, Van Rompay L, Temmerman L, Van Sinay E, Beets I, Janssen T, Husson SJ, Schoofs L 2012 Neuropeptide GPCRs in *C. elegans*. Front. Endocrinol. 3, 167 (10.3389/fendo.2012.00167) PMC3527849 23267347

[61] Frooninckx L et al. 2012 Neuropeptide GPCRs in *C. elegans*. Front. Endocrinol. 3, 167 (10.3389/fendo.2012.00167) PMC3527849 23267347

