# Supplementary material for: Neuropeptides encoded by nlp-49 modulate locomotion, arousal and egg-laying behaviours in Caenorhabditis elegans via the receptor SEB-3
Source: Philos Trans R Soc Lond B Biol Sci. 2018 Sep 10;373(1758):20170368. doi: 10.1098/rstb.2017.0368 (PMC6158228; doi:10.1098/rstb.2017.0368)
Supplement: Supplementary information [file rstb20170368supp1.pdf]

Neuropeptides encoded by *nlp-49* modulate locomotion, arousal and egg-laying behaviours in *C. elegans* via the receptor SEB-3

Yee Lian Chew, Laura J Grundy, Andre EX Brown, Isabel Beets and William R Schafer

Supplementary material

Figure S1: Unsupervised tracking of *nlp-49* and *seb-3* deletion mutants show similarities in locomotor profiles. A) Representative traces for wild-type, *nlp-49(-)* and *seb-3(-)* animals showing the midbody bend angle for all frames in a 15-minute recording (30 frames per second). B) Body bend features compared between all genotypes tested. i) Eigen Projection 2, ii) Eigen Projection 3. Eigen Projection 2 and 3 refer to the variance in sine and cosine waves, respectively, encoding the travelling wave during crawling (18, 58). Error bars indicate mean ± SD. Statistical tests: one-way ANOVA, Holm-Sidak's post-test. p-values indicated by ns = not significant, *<0.05, **<0.01, ***<0.001 and ****<0.0001. See also **Figure 2**.

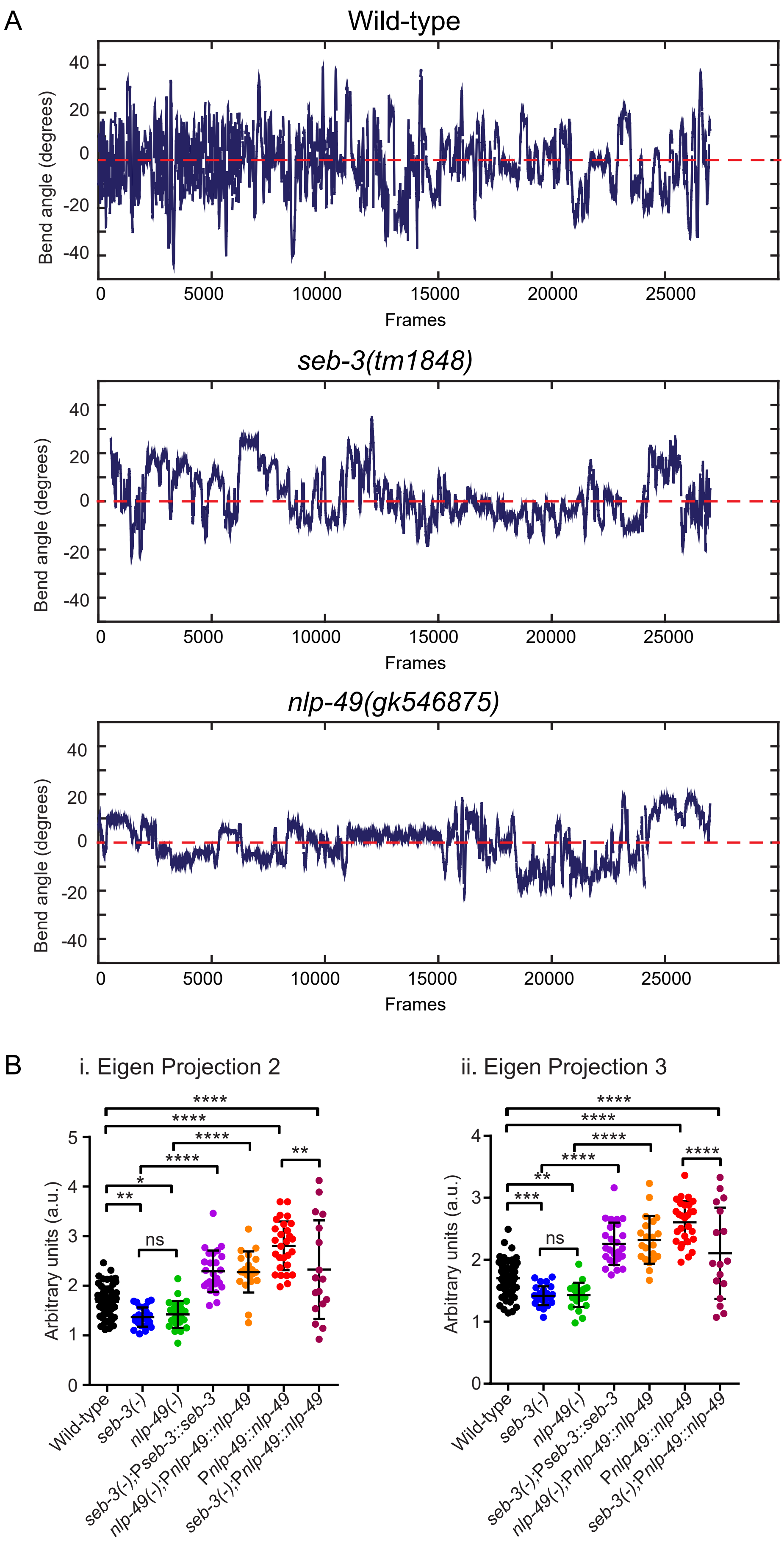
A
Wild-type
Bend angle (degrees)
Frames
seb-3(tm1848)
nlp-49(gk546875)
B
i. Eigen Projection 2
ii. Eigen Projection 3
Arbitrary units (a.u.)
Wild-type
seb-3(-)
nlp-49(-)
seb-3(-);Pseb-3::seb-3
nlp-49(-);Pnlp-49::nlp-49
Pnlp-49::nlp-49
seb-3(-);Pnlp-49::nlp-49
ns
****
***
**
*

Figure S2: *nlp-49* and *seb-3* deletion mutants show increased egg-laying compared with wild-type. A) Raster plots for three representative wild-type (grey), *seb-3(-)* (blue), and *nlp-49(-)* (green) animals indicating all egg-laying events (vertical dashes) in a 6-hour period. B) Eggs retained in uterus for wild-type animals and *nlp-49(-)* mutants expressing either a rescue transgene for *nlp-49* expression under its own promoter or a control transgene expressing *mKate2*. Compared with animals expressing a control *mKate2* transgene, animals over-expressing *nlp-49* showed significantly more eggs retained in the uterus. This was not significantly attenuated with the addition of the *nlp-49(gk546875)* mutation, most likely to due to an over-expression effect resulting from the high-copy transgenic array for P*nlp-49::nlp-49*. Eggs were counted blind to the genotype of the strains 16 hours post L4-moult. n>3 replicates, n=15 per replicate. The box-and-whisker plot shows the box extending from 25th to 75th percentiles and the line in the middle indicating the median. Whiskers extend from min to max. C) Eggs laid in liquid (average for 10 worms) for wild-type, *nlp-49(-)*, *seb-3(-)* and *nlp-49(-);seb-3(-)* double mutants. For B-C: Error bars indicate mean ± SEM. Statistical tests: one-way ANOVA, Sidak's post-test. p-values indicated by ns = not significant, **<0.01, ****<0.0001. See also **Figure 4**.

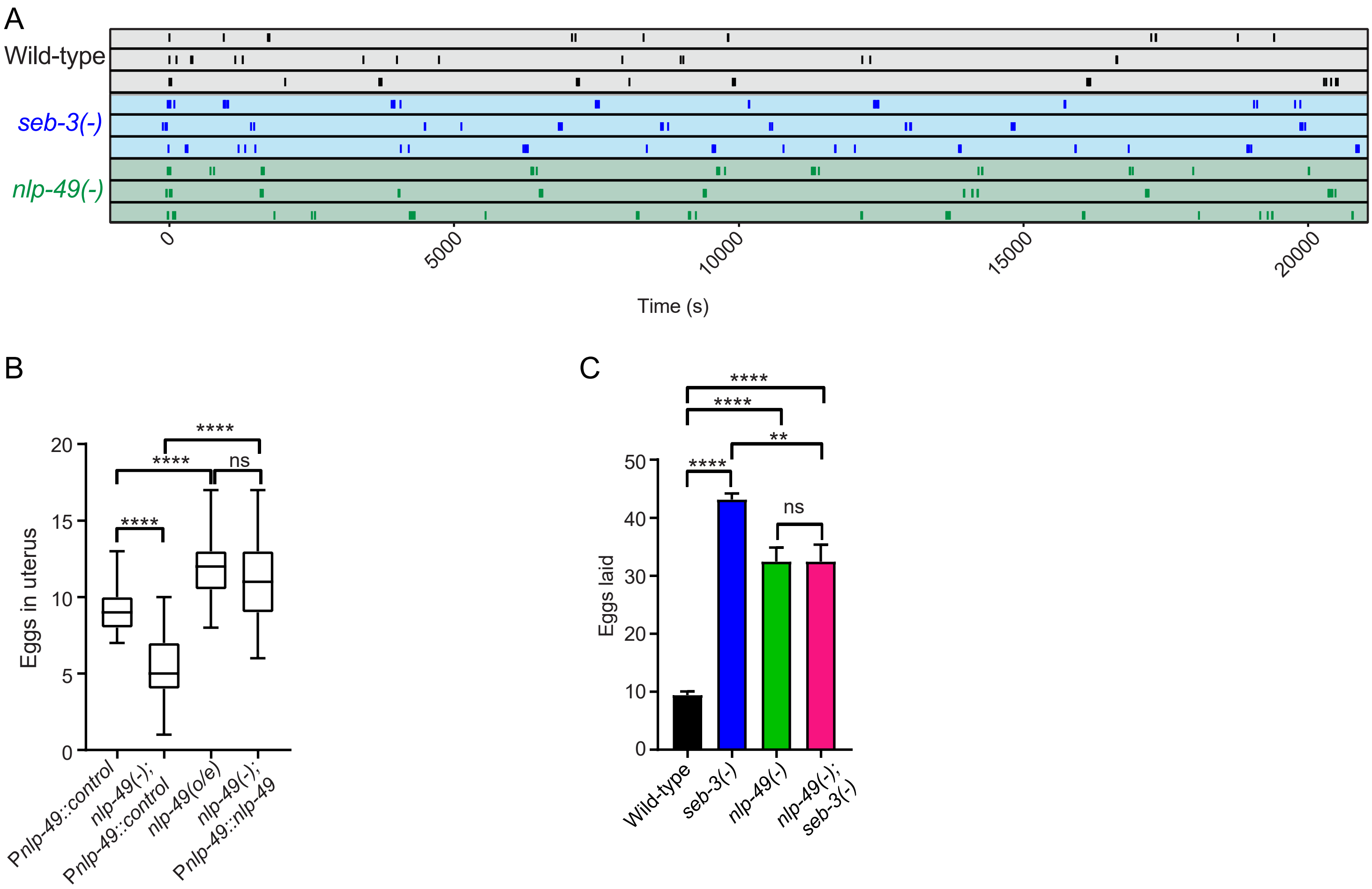
A
Wild-type
seb-3(-)
nlp-49(-)
0
5000
10000
15000
20000
Time (s)
B
Eggs in uterus
20
15
10
5
0
****
ns
Pnlp-49::control
nlp-49(-); Pnlp-49::control
nlp-49(o/e)
nlp-49(-); Pnlp-49::nlp-49
C
Eggs laid
50
40
30
20
10
0
**
Wild-type
seb-3(-)
nlp-49(-)
nlp-49(-); seb-3(-)

Table S1: Strain list. For transgenic lines, the number following the transgene refers to the concentration injected in ng/µL.

**Strain number**	**Genotype**	**Experiment**
MT2426	*goa-1(n1134)* I	Egg-laying
AQ3631	*seb-3(tm1848)* outcrossed 4x	Egg-laying, tap arousal, locomotion tracking
AQ3644	*nlp-49(gk546875)* outcrossed 6x	Egg-laying, tap arousal, locomotion tracking
AQ3701	*seb-3(tm1848);nlp-49(gk546875)*	Egg-laying, tap arousal
AQ3837	*ljEx1004[Pnlp-49::Pnlp-49gDNA + UTR::SL2-mKate2(25); unc-122::gfp(50)]*	Expression, egg-laying, locomotion tracking
AQ3851	*seb-3(tm1848);ljEx1004*	Egg-laying, locomotion tracking
AQ3853	*nlp-49(gk546875);ljEx1004*	Egg-laying, locomotion tracking
AQ3919	*ljEx1060[Pflp-1::nlp-49 gDNA + UTR::SL2-mKate2 (50); unc-122::GFP(50)]*	Egg-laying, tap arousal
AQ3920	*ljEx1061[Pflp-12::nlp-49 gDNA + UTR::SL2-mKate2 (50); unc-122::GFP(50)]*	Egg-laying, tap arousal
AQ3926	*seb-3(tm1848);ljEx985[Pseb-3::seb-3 gDNA::SL2-mKate2 (50); unc-122::GFP(50)]*	Expression, egg-laying, locomotion tracking
AQ3967	*seb-3(tm1848);ljEx1060*	Tap arousal
AQ3968	*nlp-49(gk546875);ljEx1060*	Egg-laying
AQ4009	*ljEx1091[Pnlp-49::mKate::gpd-2 3' UTR(25); unc-122::gfp(50)]*	Egg-laying
AQ4010	*nlp-49(gk546875);ljEx1091[Pnlp-49::mKate::gpd-2 3' UTR(25); unc-122::gfp(50)]*	Egg-laying
AQ4015	*seb-3(tm1848);ljEx1085[Pseb-3::mKate::gpd-2 3'UTR(25); unc-122::gfp(50)]*	Egg-laying
AQ4016	*seb-3(tm1848);ljEx1086[Pegl-6a::mKate::gpd-2 3'UTR(25); unc-122::gfp(50)]*	Egg-laying
AQ4017	*seb-3(tm1848);ljEx1088[Pegl-6a::seb-3 cDNA::SL2-mKate(25); unc-122::gfp(50)]*	Egg-laying
AQ4018	*seb-3(tm1848);ljEx1089[Pcat-1::seb-3 cDNA::SL2-GFP(25); unc-122::rfp(50)]*	Egg-laying
AQ4044	*ljIs152[integration of ljEx1004[Pnlp-49::nlp-49 gDNA+UTR(25);ccGFP(50)] backcrossed 5x*	Tap arousal
AQ4052	*seb-3(tm1848);ljIs152*	Tap arousal
AQ4319	*nlp-49(gk546875);ljEx1011[Pflp-1::gfp(50);unc-122::RFP(50)]*	Egg-laying
AQ4500	*seb-3(tm1848);ljEx1290[Pcat-1::gfp::gpd-2 3' UTR(25)]*	Egg-laying

Table S2: Reporter lines used for identification of *nlp-49* and *seb-3*-expressing cells.

Gene	Cell	Promoter	Reporter line	Reference
nlp-49	SMB	*flp-12*	NY2082 *ynIs82[Pflp-12::GFP]*	Kim and Li, 2004, J Comp Neurol
	AVK	*flp-1*	AQ3849 *ljEx1011[Pflp-1::gfp]*	Kim and Li, 2004, J Comp Neurol
seb-3	AWB	*str-1*	AQ3994 *ljEx1084[Pstr-1::mCherry]*	Troemel *et al.*, 1997, Cell
	AWA	*odr-10;gpa-6*	CX14887 *kyIs598 [gpa-6::GCaMP2.2b 50 ng/µL]*	Sengupta *et al.*, 1996, Cell
	OLQ	*ocr-4*	AQ2829 *ljEx421[pocr-4::YC3.60]*	Tobin *et al.*, 2002, Neuron
	IL1	*aqp-6*	AQ3541 *ljEx834 [Paqp-6::mcherry]*	Huang *et al.*,2007, Cell Physiol
	CEP	*cat-1*	AQ3375 *ljIs147[Pcat-1::YC3.60]*	Duerr *et al.*, 1999, J Neurosci
	AIA	*sra-11*	AQ2529 *ljEx286[Psra-11::YC3.60]*	Altun-Gultekin *et al.*,2001, Development
	DVA	*cho-1*	OH13470 *otIs354 [cho-1(fosmid)::SL2::YFP::H2B]*	Pereira *et al.*, 2015, eLife
	AVB	*sra-11*	AQ2529 *ljEx286[Psra-11::YC3.60]*	Altun-Gultekin *et al.*,2001, Development
	AIY	*ttx-3*	AQ2637 *ljEx354[pttx-3(int2)::YC3.60]*	Hobert *et al.*, 1997, Neuron
	HSN	*cat-1*	AQ3375 *ljIs147[Pcat-1::YC3.60]*	Duerr *et al.*, 1999, J Neurosci
	SMB	*flp-12*	NY2082 *ynIs82[Pflp-12::GFP]*	Kim and Li, 2004, J Comp Neurol
	PVP(-R)	*cho-1*	OH13470 *otIs354 [cho-1(fosmid)::SL2::YFP::H2B]*	Pereira *et al.*, 2015, eLife
	Ventral nerve cord motorneurons	*cho-1*	OH13470 *otIs354 [cho-1(fosmid)::SL2::YFP::H2B]*	Pereira *et al.*, 2015, eLife
	SDQ(L)	by anatomical position		WormAtlas
	AIN	unconfirmed		

Table S3: Raw data for tracking experiments. Data was obtained from at least 17 animals tracked on multiple days. See also Figure 2.
